# An Outbreak of Food-Borne Typhoid Fever Due to *Salmonella enterica* Serotype Typhi in Japan Reported for the First Time in 16 Years

**DOI:** 10.4269/ajtmh.15-0484

**Published:** 2016-02-03

**Authors:** Tetsuro Kobayashi, Satoshi Kutsuna, Kayoko Hayakawa, Yasuyuki Kato, Norio Ohmagari, Hideko Uryu, Ritsuko Yamada, Naoyuki Kashiwa, Takahito Nei, Akihito Ehara, Reiko Takei, Nobuaki Mori, Yasuhiro Yamada, Tomomi Hayasaka, Narito Kagawa, Momoko Sugawara, Ai Suzaki, Yuno Takahashi, Hiroyuki Nishiyama, Masatomo Morita, Hidemasa Izumiya, Makoto Ohnishi

**Affiliations:** National Center for Global Health and Medicine, Tokyo, Japan; Nippon Medical School Hospital, Tokyo, Japan; National Hospital Organization Tokyo Medical Center, Tokyo, Japan; Nihon University Hospital, Tokyo, Japan; National Institute of Infectious Diseases, Tokyo, Japan

## Abstract

For the first time in 16 years, a food-borne outbreak of typhoid fever due to *Salmonella enterica* serotype Typhi was reported in Japan. Seven patients consumed food in an Indian buffet at a restaurant in the center of Tokyo, while one was a Nepali chef in the restaurant, an asymptomatic carrier and the implicated source of this outbreak. The multiple-locus variable-number tandem repeat analysis showed 100% consistency in the genomic sequence for five of the eight cases.

## Introduction

*Salmonella enterica* serotype Typhi and *S. enterica* serotype Paratyphi A, B (tartrate negative), and C cause typhoid and paratyphoid fevers. They are characterized by relatively long incubation periods (14 days on average) and are common in tropical regions, including South and Southeast Asia.^1^ Although imported cases in nontropical countries (e.g., the United States and Japan) have been documented among returning travelers, cases attributable to domestic origin in these countries are rare given the improvements in hygiene in the past decades.^2^–^4^ For example, the last reported outbreak of typhoid fever in Japan occurred in 1998 when 26 people with no travel history to an endemic country were diagnosed with typhoid fever due to *S. enterica* Paratyphi A after food exposure at a public restaurant.^5^ Although approximately 100 cases of sporadic typhoid fever occur yearly in Japan, no outbreaks have been reported since the 1998 outbreak.^6^,^7^ However, this article presents a recent domestic typhoid fever outbreak in Japan caused by *S. enterica* Typhi.

## Cases

Seven people without travel history to an endemic region were diagnosed with typhoid fever between September and October 2014. Each patient diagnosed with typhoid and/or paratyphoid fever was to be reported to a local health center for government surveillance, where each patient was interviewed for history of travel and meal. All seven patients had consumed food in an Indian buffet, including curry with rice, tandoori chicken, and fresh vegetables, at a restaurant in the center of Tokyo. Patients 1–3 sat together and ate the delivered meal of the same menu separately served on plastic plates using separate spoons and forks on the same day (August 8, 2014). Patients 4–7 were exposed at the restaurant but on different dates as follows: patients 6 and 7 on August 8, 2014; patient 4 on August 22, 2014; and patient 5 on August 14, 2014. All the seven employees (four chefs and three waiters) of the Indian buffet were asymptomatic.^8^ Urine and stool samples from the seven employees were tested for chronic carriage. One employee, a Nepali chef in the restaurant (index patient 8), was the only carrier and the implicated source of this outbreak. The restaurant was not equipped with hand sterilizers, and the employees (including the index patient) did not routinely perform hand washing before starting food processing. The index patient mainly dealt with fresh salad in the restaurant. For patients 1–4 and 8, the disease was confirmed and treated at the National Center for Global Health and Medicine, whereas for patients 5–7, the disease was confirmed and treated at National Hospital Organization Tokyo Medical Center, Nippon Medical School Hospital, and Nihon University Hospital, respectively. From the outbreak epicenter, all four facilities are reachable by commuter trains. All the patients received antimicrobials from the day of diagnosis (with blood culture positivity).

None of the seven patients had a travel history to an endemic country, except patient 1, who had traveled to Nepal 6 months earlier but demonstrated no symptoms of typhoid fever on return. The index patient moved from Nepal to Japan in January 2013 and has frequent travel history to Nepal, the last time of which was in February 2014. Each of the eight patients had not had any personal contacts with the others, except patients 1–3, who worked together at a television studio on the day of the exposure to the delivered food. None of the patients had any underlying illnesses. The incubation period ranged from 10 to 27 days (mean, 17.4 days). Fever was documented in all seven patients (100%), diarrhea in six (86%), abdominal pain in two (29%), and vomiting in two (29%). *Salmonella enterica* Typhi was isolated from blood culture samples in all seven patients. The strain showed susceptibility to ampicillin, ceftriaxone, azithromycin, meropenem, and trimethoprim/sulfamethoxazole. The strain also showed intermediate susceptibility to ciprofloxacin on an E-test (minimum inhibitory concentration, 0.25 g/mL). However, the strain showed resistance to quinolones (nalidixic acid; minimum inhibitory concentration, ≥ 64 g/mL). All the patients, except patient 3, who received levofloxacin also received ceftriaxone either alone or in combination with other drugs (e.g., carbapenems and fluoroquinolones).

Two patients had a relapse. Patient 3 relapsed 40 days after completing the prescribed treatment (oral levofloxacin for 6 days, administered at a local clinic) and patient 7 relapsed 29 days after completing the treatment (intravenous ceftriaxone and oral tosufloxacin for 14 days). Both of these patients were cured after a second course of antibiotics. The index patient's stool sample showed a positive culture for *S. enterica* Typhi with the same sensitivity patterns as those in patients 1–7. Although he was asymptomatic, he received 20 days of oral moxifloxacin therapy. A stool sample taken 2 weeks after the initiation of antibiotics showed a negative culture. A summary of the clinical course, treatment, and outcomes in each patient is shown in Tables 1 and 2.

**Figure 1.**
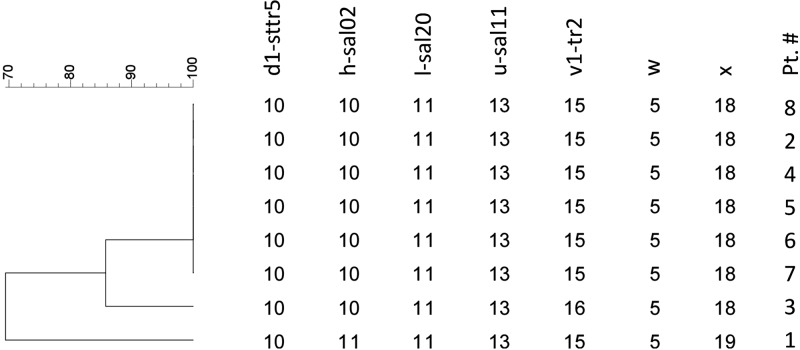
The multiple-locus variable-number tandem repeat analysis from eight patients in the outbreak of food-borne typhoid fever in Tokyo, 2014. *Salmonella enterica* Typhi isolates from all eight patients, except isolates of patients 1 and 3, had the same repetition patterns in seven loci. The isolates in patients 1 and 3 showed double-locus variants in loci SAL02 and TR4699 and single-locus variants in locus TR2, respectively.

Blood cultures of the samples from all eight patients were examined by using a multiple-locus variable-number tandem repeat analysis. The test results showed that the *S. enterica* Typhi isolates from all eight patients, except those from patients 1 and 3, had the same repetition pattern in seven loci. The isolates in patients 1 and 3 showed double-locus variants in loci SAL02 and TR4699 and single-locus variants in locus TR2, respectively (Figure 1

). The variants in both isolates had only a single tandem repetition difference as shown in the figure.

## Discussion

We believe that the fresh salad handled by the index patient, a Nepali chef in the restaurant, was the source of this outbreak for two reasons. First, the index patient mainly dealt with fresh salad without proper hand-cleaning process, and the salad was the only unheated product common to all the seven patients' menus. Second, none of the other patients, except patient 1, had a travel history to endemic regions. Patient 1 only had a travel history to Nepal 6 months earlier without presentation of typhoid fever during the trip, and the disease onset was 14 days after the exposure to the delivered food that was contaminated (compatible with the incubation period in most cases).

Although the multiple-locus variable-number tandem repeat analysis showed discrepancies in the seven loci tested for patients 1 and 3, the differences were trivial (double-locus variants in loci SAL02 and TR4699 in patient 1 and single-locus variants in locus TR2 in patient 3). As shown in a previous study, long-term carriers may excrete *S. enterica* Typhi with genetic variations because of chromosomal rearrangements, and this exceptionally low degree of variation in this outbreak indicates that the outbreak originated from the same isolate, the isolate in patient 8.^9^,^10^ In addition, it is epidemiologically evident that these patients were exposed to the same source of infection given that they sat with patient 2 and consumed the same contaminated food on the same date.

Furthermore, different dates of exposure for patients 4 and 5 indicate that the contaminated food might have been served at the same restaurant continuously between August 8 and 22, 2014. This may also indicate that other cases of typhoid fever have not been reported. In fact, 14 patients with definite diagnosis, including the seven cases in our report, were eventually documented in the local health department.^8^

The number of reported cases of typhoid fever in Japan because of *S. enterica* Typhi or *S. enterica* Paratyphi A has decreased considerably since the end of World War II (from 68,000 cases in 1945 to less than 200 cases in 1987).^4^ Since 1994, fewer than 100 cases have been reported each year, and approximately 90% of these cases are imported cases, most of which originating in South and Southeast Asia.^6^,^7^ The remaining 10% are domestic and sporadic, with the exception of outbreaks in 1993 (50 cases because of *S. enterica* Typhi), 1994 (34 cases because of *S. enterica* Paratyphi A), and 1998 (26 cases because of *S. enterica* Paratyphi A).^5^,^11^ However, as the number of restaurants serving the cuisine of other countries has dramatically increased in the past few decades, so has the proportion of immigrants from endemic countries who handle foods in these restaurants. Although advances in hygiene practices and sanitization methods have led to a tremendous decrease in domestic typhoid fever cases, the present case emphasizes the possibility of domestic outbreaks due to insufficient hand-cleaning procedures by asymptomatic food service workers from endemic countries. Given the increase in the number of asymptomatic carriers in Japan, public health officials should be aware of the potential for domestic outbreaks in the future.

The limitation of this study is that we could not include all the 14 documented cases in our report. However, 13 of the cases of typhoid fever involved in this outbreak had the same phage type, including the seven cases in this report, as described elsewhere.^8^

In conclusion, it is important to emphasize proper hygiene and sanitization practices for all food service workers, and public health safety officials should ensure that these guidelines are followed.

## Figures and Tables

**Table 1:** Food-borne outbreak of typhoid fever due to *Salmonella enterica* Typhi in Tokyo, Japan, in 2014: patient summaries

Patient no.	Age (years), sex	Past medical history	Date of exposure	Date of onset (incubation period)
1	23, F	None	August 8, 2014	August 22, 2014 (14 days)
2	39, M	None	August 8, 2014	August 25, 2014 (17 days)
3	35, M	None	August 8, 2014	August 27, 2014 (19 days)
4	5, F	None	August 22, 2014	September 7, 2014 (16 days)
5	47, M	None	August 14, 2014	August 24, 2014 (10 days)
6	26, F	None	August 8, 2014	September 4, 2014 (27 days)
7	9, M	None	August 8, 2014	August 27, 2014 (19 days)
8	31, M	Asymptomatic cholelithiasis	Not applicable	Not applicable

F = female; M = male.

**Table 2:** Food-borne outbreak of typhoid fever due to *Salmonella enterica* Typhi in Tokyo, Japan, in 2014: symptoms and clinical courses

Patient no.	Symptoms	Initiation of treatment (days after onset)	Treatment	Outcome
1	Diarrhea, fever	September 1, 2014 (10 days)	CTRX: 2 g q24h, 14 days	Cured
2	Diarrhea, fever	September 2, 2014 (8 days)	CTRX: 2 g q24h, 14 days	Cured
3	Diarrhea, vomiting, and fever	August 27, 2014 (0 day)	LVFX: 500 mg qd, 6 days	Relapsed on October 11, 2014, retreated with CTRX: 2 g q24h for 11 days and cured
4	Diarrhea, vomiting, and fever	September 12, 2014 (5 days)	CTRX: 60 mg/kg q12h, 14 days	Cured
5	Fever, abdominal pain	September 18, 2014 (25 days)	MEPM: 1 g q8h, 7 days, and then CTRX: 2 g q24h, 7 days	Cured
6	Diarrhea, fever, and sore throat	September 4, 2014 (0 day)	CPFX: 300 mg q12h + CTRX: 2 g q12h, then CTRX: 2 g q12h for a total of 14 days	Cured
7	Diarrhea, fever, right upper abdominal pain, and consciousness disorder	September 1, 2014 (5 days)	CTRX: 90 mg/kg q12h + TFLX: 300 mg qd, 14 days	Relapsed on October 15, 2014, treated with TFLX: 300 mg qd for 5 days and AZM: 300 mg qd for 5 days, and cured
8	None	September 17, 2014	MFLX: 400 mg qd, 20 days	Quit oral MFLX because of incompliance, but negative stool culture of *S. enterica* Typhi was confirmed on October 30, 2014

CTRX = ceftriaxone; LVFX = levofloxacin; MEPM = meropenem; MFLX = moxifloxacin; TFLX = tosufloxacin.

## References

[1] Basnyat B, Maskey AP, Zimmerman MD, Murdoch DR (2005). Enteric (typhoid) fever in travelers. Clin Infect Dis.

[2] Olsen SJ, Bleasdale SC, Magnano AR, Landrigan C, Holland BH, Tauxe RV, Mintz ED, Luby S (2003). Outbreaks of typhoid fever in the United States, 1960–99. Epidemiol Infect.

[3] Loharikar A, Newton A, Rowley P, Wheeler C, Bruno T, Barillas H, Pruckler J, Theobald L, Lance S, Brown JM, Barzilay EJ, Arvelo W, Mintz E, Fagan R (2012). Typhoid fever outbreak associated with frozen mamey pulp imported from Guatemala to the western United States, 2010. Clin Infect Dis.

[4] Kawamura M, Shibata M, Takahashi M, Yokoyama K, Matsushita S, Nakama A, Kai A (2009). Serovar distribution of *Salmonella* isolated from domestic and imported cases from 2000 to 2008 in Tokyo. Ann Rep Tokyo Metr Inst Pub Health.

[5] Yoda K, Koiwai K (1999). Paratyphoid fever outbreak in Chiba prefecture. Infect Agents Surveill Rept.

[6] Saito T, Shimada C, Yahata Y, Sunagawa T, Oishi K, Morita M, Izumiya H, Ohnishi M (2013). Typhoid fever in 2013. Infect Agents Surveill Rept.

[7] National Institute of Infectious Diseases (2014). IDWR surveillance data week 52, 2014. Infect Dis Wkly Rept Jpn.

[8] National Institute of Infectious Diseases (2015). Domestic Typhoid fever due to fresh salad. Infect Agents Surveill Rept.

[9] Octavia S, Lan R (2009). Multiple-locus variable-number tandem repeat analysis of *Salmonella enterica* serovar Typhi. J Clin Microbiol.

[10] Chiou CS, Wei HL, Mu JJ, Liao YS, Liang SY, Liao CH, Tsao CS, Wang SC (2013). *Salmonella enterica* serovar Typhi variants in long-term carriers. J Clin Microbiol.

[11] Kenji H, Haruo W (2002). Typhoid fever and Paratyphoid fever. Infect Agents Surveill Rept.

